# Characterization of a Novel Quorum-Quenching Bacterial Strain, *Burkholderia anthina* HN-8, and Its Biocontrol Potential against Black Rot Disease Caused by *Xanthomonas campestris* pv. *campestris*

**DOI:** 10.3390/microorganisms8101485

**Published:** 2020-09-27

**Authors:** Tian Ye, Wenping Zhang, Zhixuan Feng, Xinghui Fan, Xudan Xu, Sandhya Mishra, Lianhui Zhang, Shaohua Chen

**Affiliations:** 1 State Key Laboratory for Conservation and Utilization of Subtropical Agro-bioresources, Guangdong Province Key Laboratory of Microbial Signals and Disease Control, Integrative Microbiology Research Centre, South China Agricultural University, Guangzhou 510642, China; 20182047012@stu.scau.edu.cn (T.Y.); 20191047008@stu.scau.edu.cn (W.Z.); zhixuan.feng@mail.mcgill.ca (Z.F.); fxhscau@163.com (X.F.); xuxudan@stu.scau.edu.cn (X.X.); sandhyamanshi@gmail.com (S.M.); lhzhang01@scau.edu.cn (L.Z.); 2 Guangdong Laboratory for Lingnan Modern Agriculture, Guangzhou 510642, China

**Keywords:** DSF, biocontrol, quorum quenching, *Burkholderia anthina*, *Xanthomonas campestris* pv. *campestris*

## Abstract

Diffusible signal factor (DSF) is a type of *cis* unsaturated fatty acid, with a chemical structure of 11-methyl-2-dodecylene acid. DSF is widely conserved in a variety of Gram-negative bacterial pathogens and is involved in the regulation of pathogenic virulence. Quorum quenching (QQ) is a promising strategy for preventing and controlling quorum sensing (QS)-mediated bacterial infections by interfering with the QS system of pathogens. In this study, a novel DSF-degrading bacterium, *Burkholderia anthina* strain HN-8, was isolated and characterized for its degradation ability and potential biocontrol of black rot disease caused by *Xanthomonas campestris* pv. *campestris* (*Xcc*). The HN-8 strain exhibited superb DSF degradation activity and completely degraded 2 mM DSF within 48 h. In addition, we present the first evidence of bacterium having a metabolic pathway for the complete degradation and metabolism of DSF. Analysis of DSF metabolic products by gas chromatography–mass spectrometry led to the identification of dodecanal as the main intermediate product, revealing that DSF could be degraded via oxidation–reduction. Furthermore, application of strain HN-8 as a potent biocontrol agent was able to significantly reduce the severity of black rot disease in radishes and Chinese cabbage. Taken together, these results shed light on the QQ mechanisms of DSF, and they provide useful information showing the potential for the biocontrol of infectious diseases caused by DSF-dependent bacterial pathogens.

## 1. Introduction

Diffusible signal factor (DSF), a signal molecule produced by *Xanthomonas campestris* pv. *campestris* (*Xcc*), is a type of *cis* unsaturated fatty acid with a chemical structure of 11-methyl-2-dodecylene acid [1]. *Xcc* can cause black rot disease in cruciferous vegetables, which is a global and destructive disease, generally leading to a serious decline in the quality and yield of cruciferous crops [2,3]. This disease usually occurs in warm and humid climates, leading to V-shaped lesions on the leaf margins in crucifers [4,5]. During a severe black rot disease epidemic, the pathogen quickly disseminates and causes black rot of young stems and leaves [6]. The guttation droplets containing *Xcc* can spread across the field to infect the crown and root of healthy plants by means of flying insects, rain, wind, and water splashes under humid conditions [7]. Irreversible black rot disease commonly develops rapidly, bringing about death of crops. Black rot caused by *Xcc* is one of the most prevalent diseases in the *Brassicaceae* family around the world, which occurs heavily each year [8,9].

To control and prevent black rot disease, agricultural and chemical measures, including strengthening field management, selecting resistant varieties, and using of pesticides, are usually taken. However, among these strategies, field management is hard to strengthen because of certain limitations of agricultural areas and the long-term survival of pathogens resistant to extreme conditions, such as dryness and low temperature [10]. Moreover, common management relies on the use of pesticides on seeds or at the seedling stage, leading to pesticide residues that can affect the environment and human health, or alter the resistance of organisms in the environment [11,12,13]. Therefore, there is an urgent and crucial need to find an efficient, economic, and safe preventive approach for tackling black rot disease. Biocontrol is becoming a sustainable and promising method for efficient protection from plant diseases. In recent years, quorum quenching (QQ) based on quorum sensing (QS) has been adopted as a less toxic and potential biocontrol approach for plant protection due to its relationship with pathogenic multi-antibiotic-resistant bacteria [14,15].

QS plays an important role in bacterial adaptation in harsh environments. In addition, QS can also regulate functions related to pathogenicity [16,17]. Through the QS system, microorganisms evaluate their cell density, above a threshold level, and trigger collective developmental changes through synthesis, detection, and response to chemical signals known as autoinducers (AIs) [18,19]. Hence, disruption of the QS system, called QQ, could have a significant effect on the control of plant diseases [20].

QQ possesses two main mechanisms; one is based on QS signal molecule inhibitors (QSIs), and the other is based on QS signal molecule degradation enzymes [21,22]. The mechanism of QSIs is to prevent signal molecules from combining with receptor proteins to interfere with QS; halogenated furan ketone produced by *Delisea pulchra* was the first natural QSI to be identified [23]. For example, several studies have revealed that this compound reduces *Vibrio* infection of rainbow trout [24,25]. Many QSIs compounds have been found in natural sources including plants, animals, marine organisms, prokaryotic organisms, food, and fungi [26,27,28,29,30]. Moreover, several QS inhibition strategies have been obtained by chemically synthesizing compounds to target the biosynthetic pathways of QS. For example, eleven QS antagonists were synthesized by Kim et al., and they found that ten new analogs of *N*-acyl-homoserine lactone (AHL) or *N*-Sulfonyl homoserine lactone derivatives were shown to act as antagonists against AHL [31]. However, this compound has a certain toxicity, which limits its wide application. Another mechanism is to reduce signal molecules by producing degrading enzymes to achieve QQ. Up until now, QQ degrading bacterial enzymes obtained from microorganisms are mainly AHLs [32,33]. Overall, compared to inhibitors of signal molecules, QQ bacteria or QQ enzymes can avoid or at least reduce the selective pressure of plant pathogens entering the cell. With further research, QQ based on degrading enzymes is expected to break the bottleneck of traditional chemical control [33]. Hence, in the biocontrol of black rot disease caused by *Xcc*, the use of DSF degrading bacteria is an environmentally friendly, residue-free, and effective method. In addition, DSF has been found exist in many Gram-negative bacteria, including *Burkholderia* sp. and *Pseudomonas aeruginosa,* which are important human pathogens [34,35]. Moreover, DSF family including *cis*-2-dodecenoic acid (BDSF) and (2Z,3Z)-11-methyldodeca-2,5-dienoic acid (CDSF) has been identified and reported [36,37,38]. According to the literature, *Bacillus* and *Paenibacillus* have been screened for their degradation ability of DSF by Newman et al. [39]. However, there are few studies on DSF degradation strains and the mechanisms of QQ related to DSF degrading microbes for biocontrol have received less attention.

In this study, the novel isolate HN-8, identified as *Burkholderia anthina,* with excellent DSF degrading activity was screened, with the particular aim to: (1) characterize the isolate HN-8 combined with cellular morphology, physio-biochemistry features, and molecular sequences; (2) investigate the degradation capacity of DSF and the mechanisms of QQ; and (3) evaluate the biocontrol effects against *Xcc*. This study provides information to broaden the effectiveness of isolate HN-8 for exploiting its potential role as a biocontrol agent against black rot by *Xcc.*

## 2. Materials and Methods

### 2.1. Chemicals and Plants

DSF (≥99%) was purchased from Shanghai UDChem Technology Co., Ltd. (Shanghai, China) and dissolved in methanol to create a stock concentration of 100 mmol·L^−1^. Radishes (*Raphanus sativus*) and Chinese cabbage (*Brassica pekinensis*) were obtained from a nursery (Guangzhou, China), and healthy plants were selected for the biocontrol experiments.

### 2.2. Strains and Cultural Conditions

*Xanthomonas campestris* pv. *campestris* (*Xcc*) 8004 and *Escherichia coli* DH5*α* were provided by the Integrative Microbiology Research Centre, South China Agricultural University, Guangzhou, China. *Xcc* were cultured on Luria Bertani (LB) medium (NaCl 10.0 g·L^−1^, tryptone 10.0 g·L^−1^, and yeast extract 5.0 g·L^−1^) with rifampicin (30 μg·mL^−1^) at 28 °C. *E. coli* DH5*α* was cultivated on LB medium at 37 °C. The isolates grew on LB medium or mineral salt medium (MSM) ((NH_4_)_2_SO_4_ 2.0 g·L^−1^, Na_2_HPO_4_·12H_2_O 1.5 g·L^−1^, KH_2_PO_4_ 1.5 g·L^−1^, MgSO_4_·7H_2_O 0.2 g·L^−1^, CaCl_2_·2H_2_O 0.01 g·L^−1^, FeSO_4_·7H_2_O 0.001 g·L^−1^, and pH 7.2) with DSF (2 mmol·L^−1^) at 30 °C [40].

### 2.3. Isolation and Screening of DSF Degradation Strains

Soil samples were collected from the upper layer (5–10 cm) of the rice-growing and cruciferous plant-growing fields from five different farms near South China Agricultural University, Guangzhou, China. All of the collected soil samples were stored at 4 °C in the laboratory. For the isolation of bacteria from soil samples, serial dilutions were performed for each soil sample as described below: 5 g of soil was added to 50 mL of sterile distilled MSM medium (with 50 μmol·L^−1^ DSF as the sole source of carbon), the suspension was shaken at 30 °C and 200 rpm for 7 days, and then a series of dilutions were carried out [41,42]. An aliquot of 100 μL of each diluted soil suspension was uniformly spread on the surface of LB medium (with agar, 10.0 g·L^−1^) and the plates were incubated at 30 °C for 1–2 days. The different colonies growing on the LB medium were transferred to another LB medium plate for their single pure colony culture. The different single colonies were stored at 4 °C on LB plate for further follow-up experiments in this research.

For the screening of the bacterial isolates, 2 mmol·L^−1^ DSF was added into MSM medium as a sole source of carbon (with 10.0 g·L^−1^ agar), and then the medium was sterilized at 121 °C for 20 min. When it had cooled to approximately 50 °C, it was completely mixed and immediately poured onto plates (approximately 20 mL/plate). The plate was evenly drawn into three parts with a marker pen. One part was inoculated with distilled water as the blank control, the other part was inoculated with *E. coli* DH5*α* (OD_600_ = 1) as the negative control, and the other part was inoculated with the DSF-degrading strain (OD_600_ = 1) as the test. An ultraviolet spectrophotometer (at 600 nm; OD_600_) was used to determine the concentrations of the strains. After 2 days of culture, the plates were taken out to observe whether there was a colorless transparent ring around the colony. Each treatment was replicated three times. The bacterial strain HN-8 was screened and selected for further studies based on its effective DSF degradation.

### 2.4. Morphological and Physio-Biochemistry Characterization of Isolated Strain HN-8

The isolate grew on LB and was incubated at 30 °C under an alternating cycle of 12 h light and 12 h darkness. The morphological characteristics of the colony, including the color, shape, size, and edge of the colony, were observed and measured using an electron microscope (BH-2 Olympus, Tokyo, Japan) and a transmission electron microscope (Hitachi, Ltd., Tokyo, Japan).

The isolate HN-8 was commissioned to the Guangdong Detection Center of Microbiology (Guangzhou, China) to detect physiological and biochemical characteristics by performing Biolog tests. The type of Biolog microplates used in analysis is GNIII.

### 2.5. Molecular Identification and Phylogenetic Analysis

The isolate was cultured in 5 mL of LB medium, and then shaken at 30 °C and 200 rpm for 12–16 h. Genomic DNA was extracted using an Easy Pure Bacteria Genomic DNA Kit (TransGen Biotech, Ltd., Beijing, China). The obtained genomic DNA was re-suspended in 20 μL of distilled water and stored at −20 °C. The 16S rDNA universal bacterial primer pairs 27 F (5′-AGAGTTTGATCCTGGCTCAG-3′) and 1492 R (5′-GGTTACCTTGTTACGACTT-3′) were used for polymerase chain reaction (PCR) amplification of the DNA in an automated thermal cycler under the following conditions: 5 min of denaturation at 94 °C, followed by 32 cycles at 94 °C for 30 s, 55 °C for 30 s, and a final extension at 72 °C for 1 min [43,44]. The purified PCR product was sequenced by Genewiz Co., Ltd. (Suzhou, Jiangsu Province, China).

The sequences were analyzed by the nucleotide Basic Local Alignment Search Tool (BLASTn), compared to the genes in the GenBank database, and then deposited in GenBank (accession number: MG561937.1). Similar and relevant sequences were downloaded from the National Center for Biotechnology Information (NCBI), and were used for phylogenetic analysis and construction of the phylogenetic tree [45].

### 2.6. Antibiotic Sensitivity Test

Antibiotic sensitivity of HN-8 was also tested for further experiments. The isolate was cultured in 5 mL LB medium, and then shaken at 30 °C and 200 rpm for 12–16 h. The overnight cultures and the respective antibiotics were added to the LB medium, and then shaken at 30 °C and 200 rpm for 24 h. The antibiotics used in this test included ampicillin (AMP; 50 mg·mL^−1^), carbenicillin (CARB; 50 mg·mL^−1^), streptomycin (STR; 50 mg·mL^−1^), tetracycline (TET; 5 mg·mL^−1^), kanamycin (KAN; 50 mg·mL^−1^), gentamicin (GEN; 50 mg·mL^−1^), neomycin sulfate (NS; 20 mg·mL^−1^), rifampicin (RIF; 25 mg·mL^−1^), and chloramphenicol (CM; 30 mg·mL^−1^) at different concentrations (i.e., 5, 10, 20, 50, 150, 200, 250, 300, 350, and 400 μg·mL^−1^). An ultraviolet spectrophotometer (at 600 nm; OD_600_) was used to detect the growth of strain HN-8 with different concentrations of these antibiotics. Each treatment was replicated three times.

### 2.7. DSF Degradation Capacity Test

An ultraviolet spectrophotometer and the high-performance liquid chromatography (HPLC) technique were employed to detect the growth and degradation capacity of the isolate HN-8, respectively. A two-day-old colony of DSF-degrading isolate was added into the LB medium; the suspension was shaken at 30 °C and 200 rpm for 12–16 h, and then transferred to MSM medium (with 2 mmol·L^−1^ DSF as the sole source of carbon). The cultures were incubated at 30 °C and 200 rpm, and the same volume of cultures was taken out and centrifuged to obtain the supernatant, where the residual DSF was extracted from different intervals [46]. HPLC was performed to determine the amount of residual DSF under the following conditions: C_18_ reverse chromatographic column, flow rate of 1 mL·min^−1^, column temperature of 35 °C, mobile phase of methanol/water = 80:20 (*ν*:*ν*), detection wavelength of 210 nm, and injection quantity of 20 μL [47]. Each treatment was replicated three times.

### 2.8. Identification of DSF Degradation Products

Gas chromatography–mass spectrometry (GC–MS) (Model 7890B/5977B, Agilent Technologies, USA), which uses Agilent MassHunter Workstation software, was employed to detect the degradation products of the isolate HN-8. The HN-8 suspension, shaken at 30 °C and 200 rpm for 12–16 h, was added into the MSM medium (with 5 mmol·L^−1^ DSF as the sole source of carbon). As described and extracted above, samples were collected at regular intervals and mass spectrometry analyses were matched with authentic standard compounds from the National Institute of Standards and Technology (NIST, USA) library database. The analytical conditions for GC–MS were as follows: injection amount, 1.0 μL; injection mode into the gas chromatograph, split with a 10:1 ratio; capillary column, HP-5 MS Ultra Inert column (30 m\0.25 μm\0.25 μm); column oven temperature, 60 °C for 5 min, then an increase of 10 °C/min, followed by 300 °C for 29 min; injection port temperature, 280 °C; carrier gas, helium; analytical mode, scan mode. Mass spectra were recorded in the *m*/*z* range of 40–430 [47,48].

### 2.9. Biocontrol Assay of Strain HN-8 against Xcc

Based on previous experimental results, the DSF-degrading isolate HN-8 was used for in vitro tests of biocontrol against black rot caused by *Xcc* on radishes (*Raphanus sativus* L. var. *raphanistroides* (Makino) Makino) and Chinese cabbage (*Brassica pekinensis* (Lour.) Rupr.). The bacterial suspensions were inoculated on radish root slices containing suspensions of black rot pathogen *Xcc* (4 × 10^8^ CFU·mL^−1^), suspensions of DSF-degrading strain HN-8 (2 × 10^8^ CFU·mL^−1^), and suspensions of *Xcc* mixed with HN-8. Radish root slices inoculated only with distilled water were set as the control. Biocontrol tests were also performed on Chinese cabbage, which included four treatments, as follows: (1) cabbage treated with *Xcc* (4 × 10^8^ CFU·mL^−1^); (2) Chinese cabbage leaves treated with *Xcc* mixed with HN-8; (3) Chinese cabbage leaves treated with HN-8 (2 × 10^8^ CFU·mL^−1^); and (4) Chinese cabbage leaves treated with distilled water (the control). Each treatment was repeated three times. The treated plants were observed daily, and disease incidence and disease severity were recorded from the inoculation [16]. The details of the tests was described as follows. Healthy plants were washed with running distilled water and placed onto the surfaces of the wet sterilized filter papers on a sterilized plate. The strains used in this part were already cultured in 5 mL LB medium and shaken at 30 °C and 200 rpm for 12–16 h. Then, 100 μL of bacterial suspensions were inoculated on each plant. Next, the plates were incubated at 30 °C for 3 days in an incubator. The macerated area and macerated tissue weight were measured to evaluate the disease severity. The diameter of the macerated region (square millimeters) was calculated and scooped out to measure the weight. The percentage of maceration was calculated by comparing with the pre-inoculation tissue weight.

## 3. Results

### 3.1. Isolation and Selection of DSF-Degrading Strain HN-8

**Figure 1:**
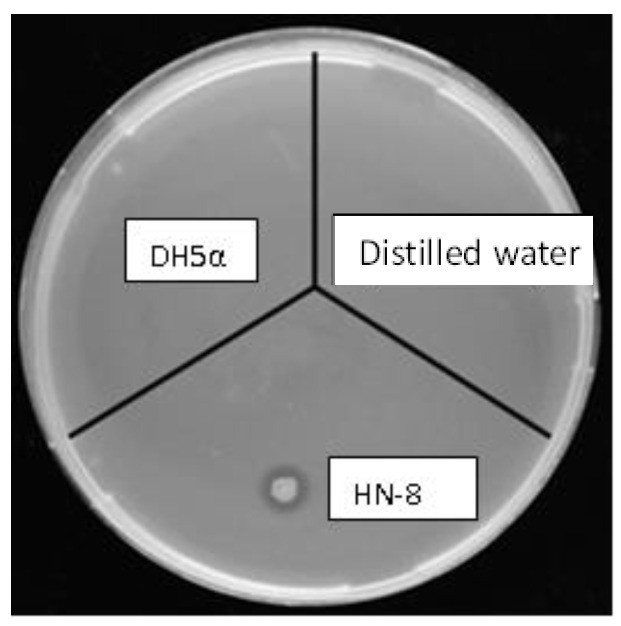
Diffusible signal factor (DSF) degradation ability test of *Burkholderia anthina* HN-8.

Four isolates with potential DSF-degrading ability were obtained from the collected soil samples. Among them, the isolate that showed the highest DSF-degrading capacity was chosen as the biocontrol agent candidate, designated as HN-8. An obvious degrading zone of isolate HN-8 inoculated on the MSM plate with DSF as the sole carbon source appeared, but no degrading zone was observed on the blank control inoculated with distilled water and the negative control inoculated with *Escherichia coli* DH5*α* (Figure 1). For confirming this phenomenon, experiments were further carried out via HPLC to detect its DSF-degrading capacity.

### 3.2. Physio-Biochemistry and Morphological Characterization of Isolate HN-8

Isolate HN-8 was cultured on an LB plate for 48 h, and its morphological characterization shows that the colony is round with neat edges, protruding and yellow in color (Figure S1). When it grew in LB liquid medium for 48 h, the culture medium showed diffuse turbidity. Microscopic observation shows that the cell morphology of the strain HN-8 is rod-shaped (Figure S2). The scanning electron microscope (SEM) observation exposed that the strain HN-8 lacks flagella (Figure S3).

The biochemical characterization (Table 1) indicate that the strain HN-8 is Gram-negative, aerobic, and motile. It is unable to utilize d-glucose, d-lactose, raffinose, sucrose, cellobiose, arabinose, rhamnose, and maltose. For the biochemical tests, including catalase, oxidase, casein hydrolysis, gelatin liquefaction, *O*-nitrophenyl-β-d-galactoside (ONPG), lysine decarboxylase, and ornithine decarboxylase, the reaction results are all positive. On the contrary, the reaction results for urease, amylolysis, nitrate reduction, hemolysis, and arginine dihydrolase are negative.

### 3.3. Molecular Identification of the Isolate HN-8

**Figure 2:**
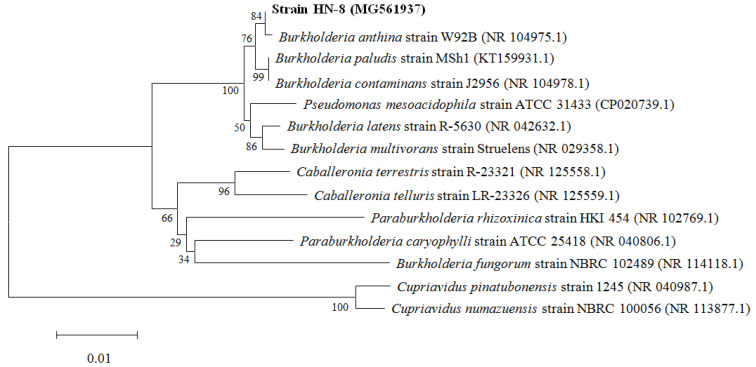
Phylogenetic tree on the basis of the 16S rRNA sequence of *Burkholderia anthina* HN-8 and related strains.

The genomic DNA of the isolate HN-8 was amplified by primer pair 27 F/1492 R, producing fragments of ~1500 bp. Sequences of the isolate HN-8 were aligned, edited, analyzed, and deposited in the GenBank database. Phylogenetic analysis of the 16S rRNA gene sequence showed that the type strain is a member of the *Burkholderia* genus, since it shares the highest sequence similarity (99%) with the 16S rRNA gene sequences of other identified *Burkholderia* spp. Additionally, the neighbor-joining analysis highlighted that the strain HN-8 shares about 99% of its identity with the corresponding sequences of *Burkholderia anthina* strain W92B (GenBank number: NR 104975.1) (Figure 2). Combined with the colony morphology, the biochemical characteristics, and the physiological and 99% homology of partial 16S rRNA, strain HN-8 was preliminarily identified as *B. anthina.* The accession number for the 16S rRNA gene sequences of the *B. anthina* strain HN-8 deposited in the GenBank database is MG561937.1.

### 3.4. Antibiotic Sensitivity

The results shown in Figure S4 indicate that the resistance of DSF-degrading strain HN-8 to AMP (50 mg·mL^−1^), CARB (50 mg·mL^−1^), and STR (50 mg·mL^−1^) reached 400 μg·mL^−1^ or above; to KAN (50 mg·mL^−1^) reached 200 μg·mL^−1^; to TET (5 mg·mL^−1^) reached 50 μg·mL^−1^; to GEN (50 mg·mL^−1^) and NS (20 mg·mL^−1^) reached 10 μg·mL^−1^; and to RIF (25 mg·mL^−1^) and CM (30 mg·mL^−1^) was less than 10 μg·mL^−1^.

The results of the antibiotic sensitivity test show that the DSF-degrading strain HN-8 has excellent antibiotic resistance, especially to ampicillin, carboxypenicillin, and streptomycin. This result will be used as a reference in future studies with appropriate antibiotics, and its resistance to many antibiotics is a great advantage to further apply strain HN-8 as a biocontrol agent.

### 3.5. DSF Degradation Kinetics

**Figure 3:**
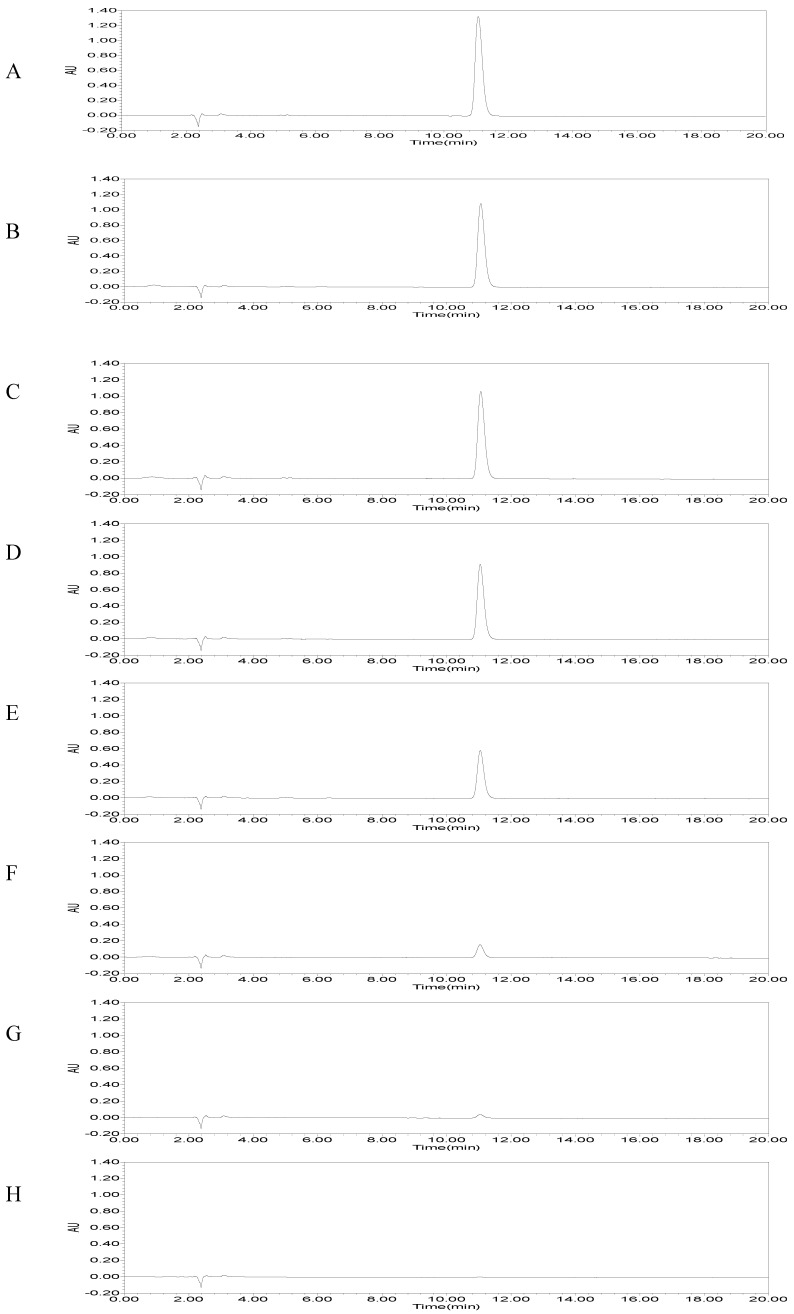
The remaining amount of DSF at different time intervals was determined by high-performance liquid chromatography (HPLC). (**A**) The mineral salt medium (MSM) with DSF alone as control. DSF degradation by the strain *Burkholderia anthina* HN-8 at 12 h (**B**), 18 h (**C**), 24 h (**D**), 30 h (**E**), 36 h (**F**), 42 h (**G**), and 48 h (**H**).

**Figure 4:**
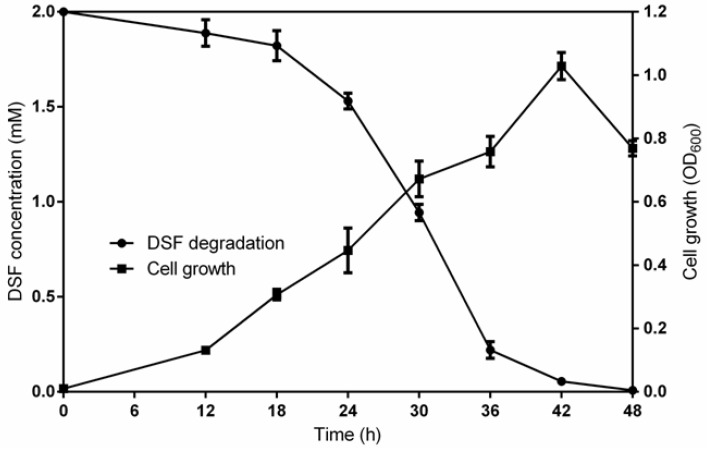
Relationship between DSF degradation and growth of *Burkholderia anthina* HN-8. Symbols: ●, DSF degradation by the strain HN-8; ■, cell growth.

Based on the results of the DSF-degrading capacity test, the potential strain grew in MSM medium and the overnight suspensions were centrifuged and extracted to detect the amount of residual DSF in the fractions. The results of HPLC revealed that the amount of DSF gradually decreased until there was none at all as time went on (Figure 3). More specifically, the DSF degradation by *B. anthina* HN-8 at 12, 18, 24, 30, 36, 42, and 48 h was 5.6%, 8.9%, 23.5%, 52.8%, 89.0%, 97.3%, and 100%, respectively (Figure 4).

As shown in Figure 4, DSF degradation was achieved with the increased growth of strain HN-8. With the gradual degradation of DSF, the growth of HN-8 also became faster and faster. In the MSM medium with DSF as the only carbon source, the logarithmic growth period of HN-8 was between 12 and 42 h, with no obvious stable period. At 42 h, only a little amount of DSF was left, and the growth of the strain decreased. Finally, the residual amount of DSF was not detectable by HPLC at 48 h post-incubation.

### 3.6. Degradation Products of DSF

In all samples, collected between 0 and 48 h, a significant compound with the molecular formula and molecular weight of C_13_H_24_O_2_ and 213, respectively, was detected and eluted at 17.614 min, with a characteristic mass fragment [M+] at *m*/*z* = 99.04 and major fragment ions at *m*/*z* = 43.06 and 212.17 (Figure S5a). This compound is the same as DSF. As the amount of DSF decreased, a new compound at a retention time (RT) of 20.052 min appeared, which was similar to dodecanal with *m*/*z* = 43.94 as the base peak (Figure S5b).

**Figure 5:**
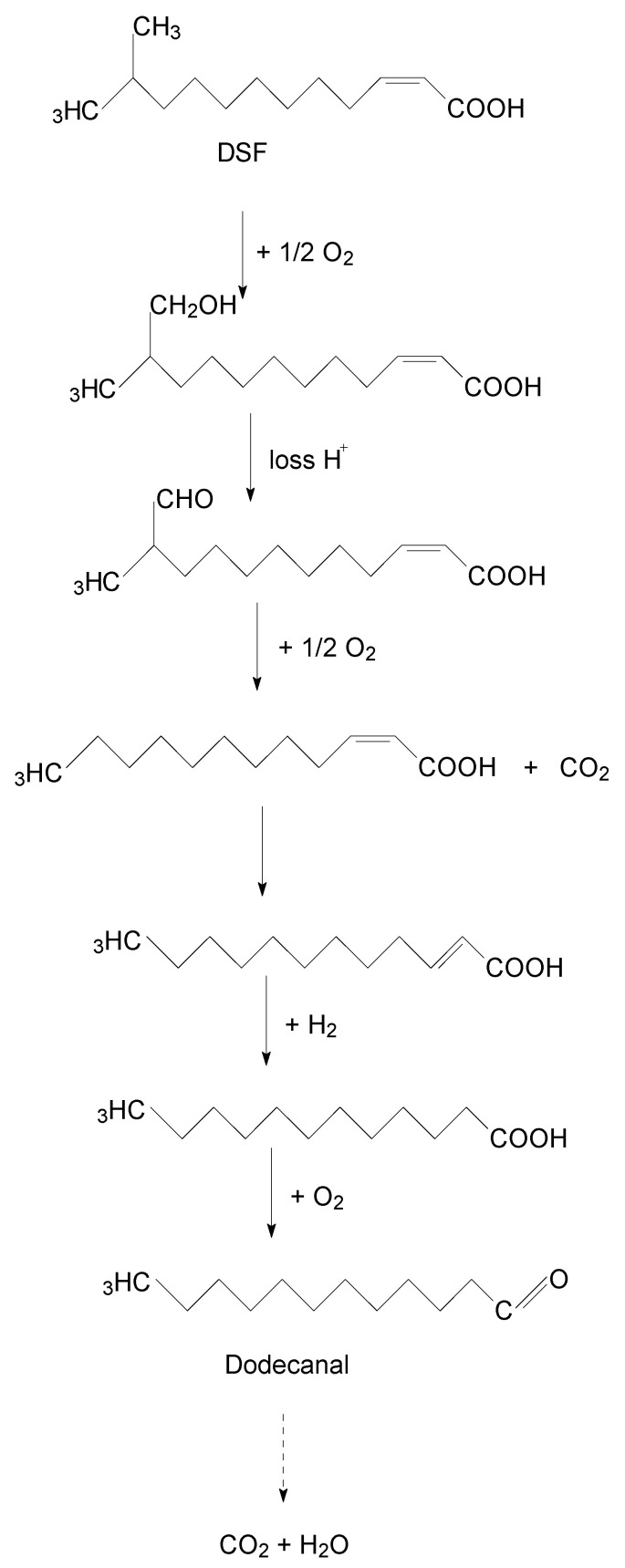
Proposed degradation pathway of DSF in *Burkholderia anthina* strain HN-8. The solid arrow means the degradation pathway inferred from the detected product dodecanal, and the dotted arrow means the undetected products.

A new degradation pathway of DSF in strain HN-8 was proposed based on the chemical structures of DSF and the metabolic product formed during the degradation process (Figure 5). First, the hydrogen on the branched carbon atoms of DSF was oxidized to hydroxyl, forming hydroxyl fatty acids. The hydroxyl fatty acids continued to oxidize and remove the carboxyl groups to form fatty acids with one less carbon atom. The shortened carbon chain continued to undergo oxidation, in which the *cis* double bond of the unsaturated fatty acid was converted to a *trans* double bond and hydrogenated to a saturated fatty acid, which was then oxidized to aldehyde. Eventually, it broke down into carbon dioxide and water.

### 3.7. Biocontrol Efficacy of Strain HN-8 against Black Rot Caused by Xcc

**Figure 6:**
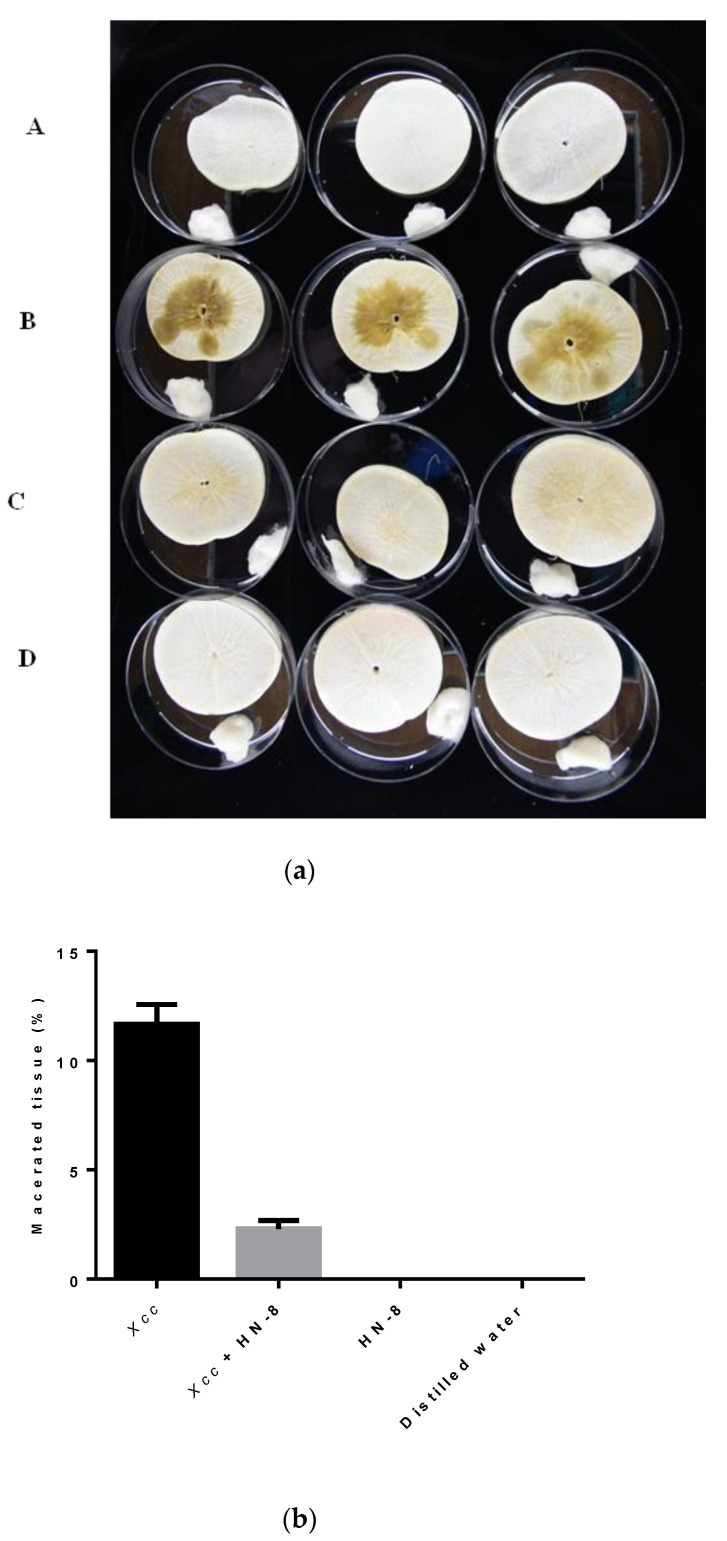
Biocontrol test of *Burkholderia anthina* HN-8 against black rot disease on radish slices under laboratory conditions. (**a**) Panel (**A**) shows radish root slices inoculated only with distilled water; panel (**B**) shows radish root slices inoculated only with the suspensions of black rot pathogen *Xcc* (4 × 10^8^ CFU·mL^−1^); panel (**C**) shows radish root slices co-inoculated with the suspensions of *Xcc* mixed with HN-8; panel (**D**) shows radish root slices inoculated only with the suspensions of DSF-degrading strain HN-8 (2 × 10^8^ CFU·mL^−1^). (**b**) Amount of macerated tissue in each treatment.

**Figure 7:**
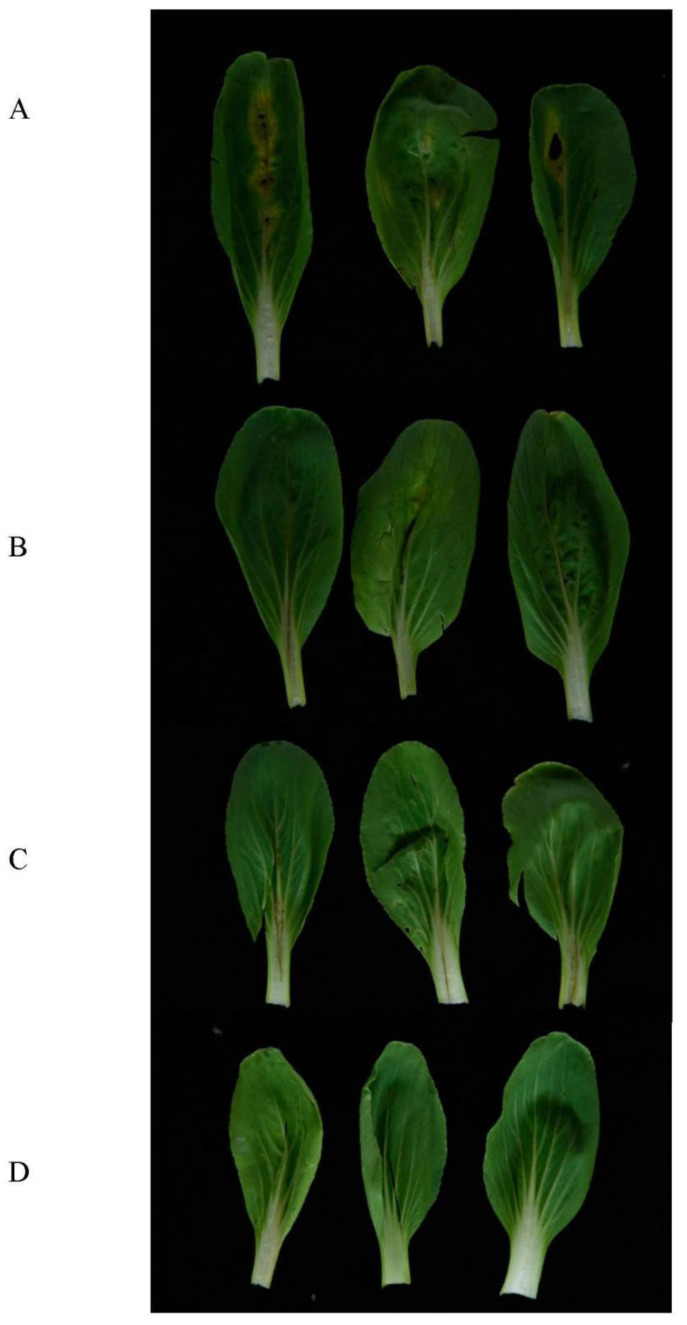
Biocontrol test of *Burkholderia anthina* HN-8 against black rot disease on Chinese cabbage under laboratory conditions. (**A**), inoculated only with the suspensions of black rot pathogen *Xcc* (4 × 10^8^ CFU·mL^−1^); (**B**), co-inoculated with the suspensions of *Xcc* mixed with HN-8; (**C**), inoculated only with the suspensions of DSF-degrading strain HN-8 (2 × 10^8^ CFU·mL^−1^); (**D**), inoculated only with distilled water.

The inoculation tests show that the *B. anthina* HN-8 significantly reduced the severity of black rot disease and it did not cause any disease symptoms in the radish roots or Chinese cabbage leaves when inoculated alone. One day after inoculation, no black rot were found in either the radish root slices or the Chinese cabbage leaves. Two days after inoculation, black rot symptoms appeared in the radish root slices and the Chinese cabbage leaves inoculated with the *Xcc* suspension and co-inoculated with *Xcc* mixed with *E. coli* DH5*α*, but no symptoms were found in the radish root slices or Chinese cabbage leaves co-inoculated with the *Xcc* mixed with HN-8. Three days after inoculation, slight symptoms were observed in the radish root slices co-inoculated with the HN-8 isolate and the *Xcc* suspension (Figure 6aC), but no symptoms were found in the Chinese cabbage leaves co-inoculated with the HN-8 isolate mixed with the *Xcc* suspension (Figure 7B). During this period, serious decay occurred in the radish root slices and the Chinese cabbage leaves inoculated with the *Xcc* suspension only (Figure 6aB or Figure 7A). Moreover, no symptoms were observed in the radish root slices or Chinese cabbage leaves inoculated only with distilled water (Figure 6aA) or Figure 7D) or with the HN-8 isolate suspension (Figure 6aD or Figure 7C) after inoculation. As shown in Figure 6b, the weight of the macerated tissue was significantly reduced at 3 days post-inoculation of strain HN-8, compared to the individual *Xcc* treatment.

## 4. Discussion

Cruciferous plants are some of the favorite and most edible vegetables consumed extensively worldwide, greatly affected by bacterial black rot disease caused by *Xanthomonas campestris* pv. *campestris* (*Xcc*) [12]. The prevention and control of black rot disease have become important concerns, due to the hugely destructive impacts of block rot disease on the productivity and quality of cruciferous plants [11]. In recent years, QQ has been increasingly interested in the development of a biocontrol method based on QS of microorganisms as an alternative and effective way to control plant diseases [16,17,47,48]. Among all of the methods of QQ, biocontrol bacteria with degrading signal molecule activity have appeared as efficient tools in plant disease management [49]. Meanwhile, many bacterial species have been used as QQ tools for the biocontrol of plant diseases, such as *Paenibacillus*, *Bacillus*, *Actinobacteria*, *Bacteroidetes*, *Lactobacillus*, *Proteobacteria*, *Firmicutes*, *Cupriavidus,* and *Acinetobacter* [39,47,48,49,50,51], demonstrating that the screening and application of QQ bacteria as biological control agents for the prevention and control of plant diseases are relatively effective. To date, many reports of QQ bacteria for plant heath management focus on AHL-mediated diseases [32,33]. Thus, in this research, a DSF-degrading strain, namely, HN-8, was successfully isolated with the capacity to rapidly degrade high concentrations of DSF in a short time and to effectively reduce black rot disease under laboratory conditions. This strain was identified as *Burkholderia anthina* based on its morphological, physiological, and biochemical characteristics, as well as 16S rRNA sequence analysis. The results indicate that *B. anthina* HN-8 has the potential to be applied as a powerful biocontrol agent against black rot in cruciferous plants.

Many plant pathogenic bacteria, including *Xcc,* regulate the expression of factors that contribute to virulence and rely on cell–cell signaling [51,52]. There are diverse structural classes of signals produced by pathogenic bacteria. The diffusible signal factor (DSF), a *cis*-unsaturated fatty acid, was first identified in the black rot disease pathogen *Xanthomonas campestris* [2,53]. Furthermore, recent work has demonstrated that DSF family signals are involved in interspecies signaling, which regulates the bacterial behavior and virulence factor [54,55,56]. Not only in plant pathogens, but also in several important human pathogens, such as *Pseudomonas aeruginosa,* structurally related molecules are produced to regulate biofilm formation, virulence, and antibiotic tolerance [57]. Degradation of these diverse signals could be a potential strategy as a biocontrol method [48]. Based on this, the DSF-degrading capacity of the novel isolated strain HN-8 with DSF-degrading activity was evaluated. The results of HPLC revealed that strain HN-8 can completely degrade DSF within 48 h. Further, these results also suggest that *B. anthina* HN-8, with the capacity to rapidly degrade high concentrations of DSF in a short time, may be involved in the biocontrol management of black rot caused by *Xcc*. To the best of our knowledge, there are few reports of DSF degradation from *B. anthina*. For the first time, this study proved that *B. anthina* can reduce the severity of black rot disease caused by *Xcc* by degrading the DSF signals.

Moreover, GC–MS was employed to detect the degradation products of the isolate HN-8, and based on the chemical structures of DSF and the metabolic products formed during degradation process, the metabolic pathway of DSF in strain HN-8 (Figure 5) was proposed as follows: firstly, the hydrogen on the branched carbon atoms of DSF was oxidized to hydroxyl, forming hydroxyl fatty acids. Secondly, hydroxyl fatty acids continued to oxidize and remove carboxyl groups to form fatty acids with one less carbon atom. Thirdly, the shortened carbon chain continued to undergo oxidation, in which the *cis* double bond of the unsaturated fatty acid was converted into a *trans* double bond and hydrogenated to a saturated fatty acid, which was then oxidized to aldehyde. Eventually, all intermediate products were transitory and faded away without any non-cleavable metabolites at the end of the experiment. This is the first report of a DSF metabolic pathway by oxidation–reduction in a microorganism, which we propose is of vital importance in DSF degradation.

In this research, the control efficiency of the strain HN-8 against *Xcc* on plants was evaluated under laboratory conditions. The inoculation tests showed that *B. anthina* HN-8 significantly reduced the severity of black rot disease in plants compared to inoculation with the *Xcc* suspension. The results also showed that the strain HN-8 could not only significantly reduce the severity of black rot disease in plants, but could also avoid causing any disease symptoms when inoculated only under laboratory conditions. The main advantages of the simplified method include (1) shortening the test cycle (2) and minimizing the interference factors during the biocontrol test. However, a biocontrol test that was carried out in vitro, on detached parts of plants, cannot be considered exhaustively to assess the activity of the antagonistic microorganisms. Therefore, the biocontrol assay in this study is a preliminary test. The biocontrol efficiency of QQ bacteria maybe not be exhibited ideally in plant tests [58,59]. In future, studying the potency of the strain HN-8 for application as a biocontrol agent against black rot disease caused by *Xcc* in cruciferous plants under field conditions would be interesting.

This research of the potential biocontrol novel isolate HN-8 was mainly focused on DSF degradation and severe black rot disease caused by *Xcc* in cruciferous plants. The strain HN-8 exhibited rapid degradation of high concentrations of DSF in a short time, which is vital to ensure an efficient prevention of pathogenic bacteria in plant assays. Additionally, the strain HN-8 had remarkable antibiotic resistance to ampicillin, carboxypenicillin, and streptomycin. The multiple beneficial abilities of the *B. anthina* strain HN-8 allows it to be regarded as an effective biocontrol agent, and to be considered for commercial development for management strategies of black rot caused by *Xcc* in cruciferous plants, similar to the *Lactobacillus plantarum* strain BY used for biocontrol of soft rot caused by *Pectobacterium carotovorum* subsp. *carotovorum* in Chinese cabbage under field conditions [50].

## 5. Conclusions

In this study, a novel QQ bacterial isolate, i.e., *B. anthina* HN-8, exhibiting excellent DSF degradation, and potent biocontrol activities against *Xcc*, was isolated and characterized. The strain HN-8 was capable of rapidly degrading DSF, and completely degraded DSF within 48 h. In addition, the application of strain HN-8 was able to significantly reduce black rot disease in radishes and Chinese cabbage. As a promising biocontrol agent, strain HN-8 could be further explored to counter other DSF-dependent bacterial pathogens such as *Pseudomonas aeruginosa*. Furthermore, strain HN-8 possesses a metabolic pathway for the complete degradation and metabolism of DSF. This is the first report of a pathway for DSF degradation by oxidation–reduction in a microorganism. These findings not only characterize a highly efficient DSF degradation bacterial isolate for biotechnological application, but they also shed light on the mechanisms of QQ in relation to DSF. In the future, further in-depth studies based on related enzymes and genes are needed to elaborate their role in the evolution of novel catabolic pathways and the molecular mechanisms of DSF degradation by strain HN-8.

## Figures and Tables

**Table 1:** Biochemical characteristics of strain HN-8.

Characteristics	Results	Characteristics	Results
Gram staining	−	Amylolysis	−
Oxidase	+	Casein hydrolysis	+
Gelatin liquefaction	+	Arginine dihydrolase	−
Ornithine decarboxylase	+	d-glucose	−
d-lactose	−	Raffinose	−
Sucrose	−	Cellobiose	−
Arabinose	−	Rhamnose	−
Maltose	−	Hemolysis	−
O-nitrophenyl-β-d-galactoside	+	Lysine decarboxylase	+

Note: +, tested positive; −, tested negative.

## Supplementary Materials

The following are available online at https://www.mdpi.com/2076-2607/8/10/1485/s1. Figure S1: Colony characteristics of the strain *Burkholderia anthina* HN-8 on LB; Figure S2: Morphological characteristics of *Burkholderia anthina* HN-8 under an electron microscope; Figure S3: Morphological characteristics of *Burkholderia anthina* HN-8 under Hitachi TEM System (6000×); Figure S4: Antibiotic sensitivity of *Burkholderia anthina* HN-8; Figure S5: Mass spectra and proposed structures of the detected degradation products appearing under gas chromatography–mass spectrometry (GC–MS). (a) DSF; (b) dodecanal.
