# Supplementary material for: Characterization of a Novel Quorum-Quenching Bacterial Strain, Burkholderia anthina HN-8, and Its Biocontrol Potential against Black Rot Disease Caused by Xanthomonas campestris pv. campestris
Source: Microorganisms. 2020 Sep 27;8(10):1485. doi: 10.3390/microorganisms8101485 (PMC7601453; doi:10.3390/microorganisms8101485)
Supplement: Supplementary file 1 [file microorganisms-08-01485-s001.pdf]

Supplementary materials

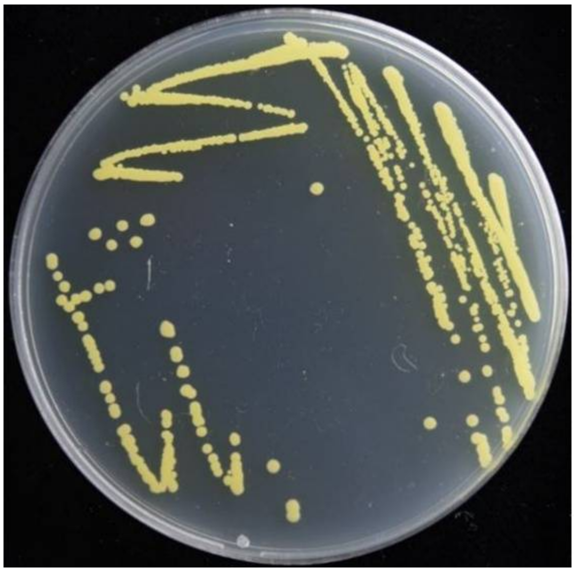

Figure S1 Colony characteristics of the strain *Burkholderia anthina* HN-8 on LB.

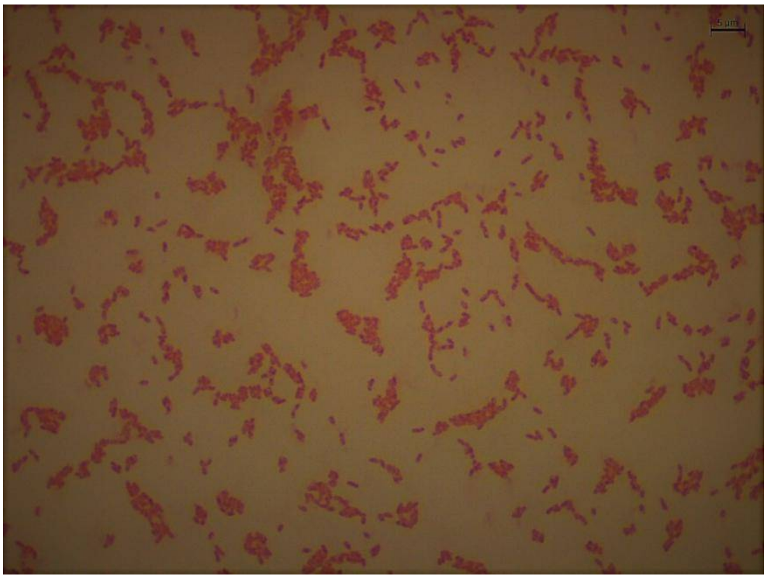

Figure S2 Morphological characteristics of *Burkholderia anthina* HN-8 under an electron microscope.

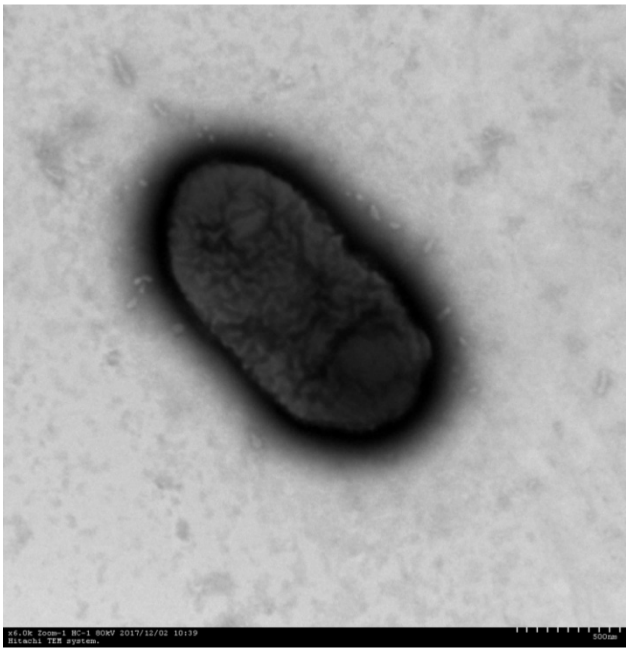

Figure S3. Morphological characteristics of *Burkholderia anthina* HN-8 under Hitachi TEM System (6000×).

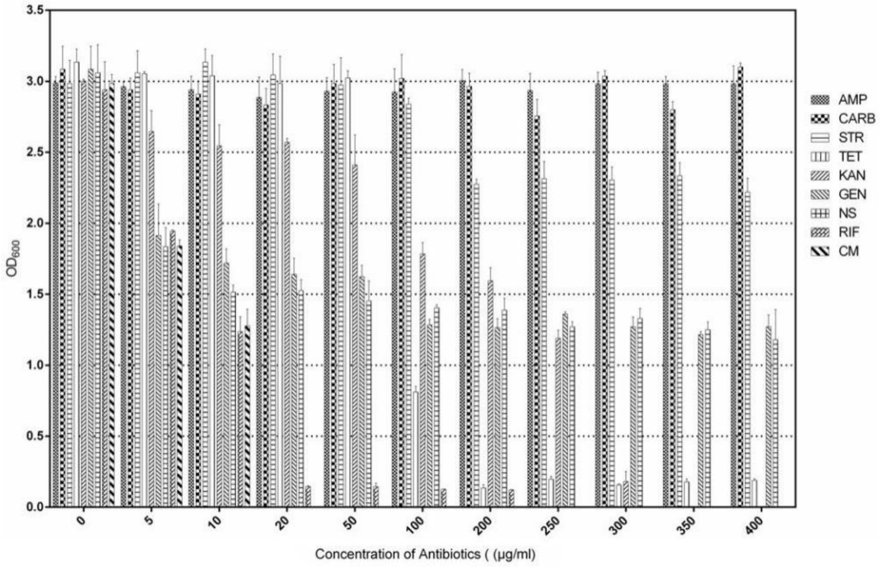

Figure S4. Antibiotic sensitivity of the *Burkholderia anthina* HN-8.

a

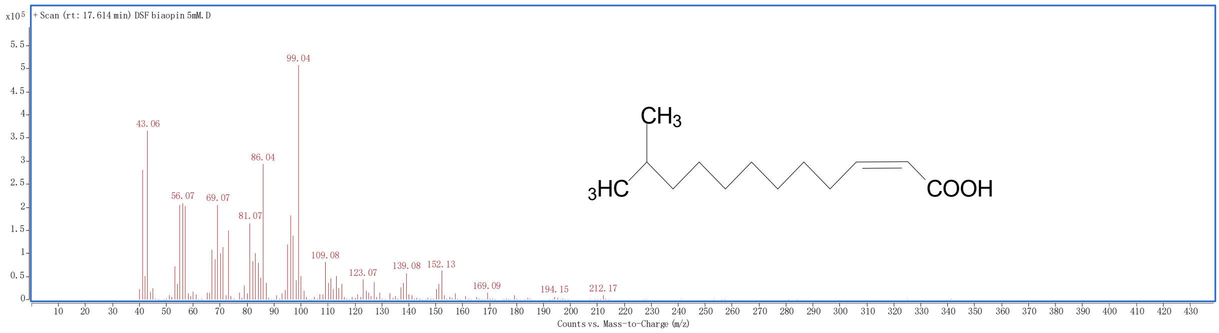

b

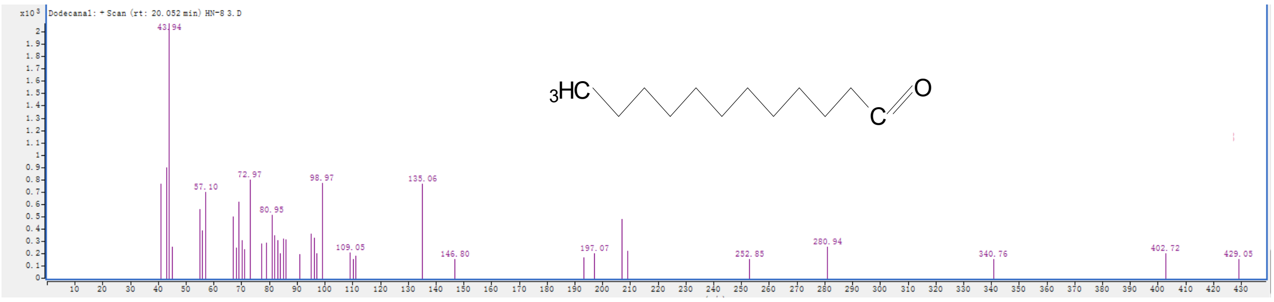

Figure S5. Mass spectra and proposed structures of the detected degradation products appearing under gas chromatography–mass spectrometry (GC–MS). (a) DSF; (b) dodecanal.
